# The inhibitory effect of LPS on the expression of GPR81 lactate receptor in blood-brain barrier model in vitro

**DOI:** 10.1186/s12974-018-1233-2

**Published:** 2018-07-04

**Authors:** Elizaveta B. Boitsova, Andrey V. Morgun, Elena D. Osipova, Elena A. Pozhilenkova, Galina P. Martinova, Olga V. Frolova, Raissa Ya Olovannikova, Abolghasem Tohidpour, Yana V. Gorina, Yulia A. Panina, Alla B. Salmina

**Affiliations:** 1 Research Institute of Molecular Medicine and Pathobiochemistry, Krasnoyarsk State Medical University named after Prof. V.F. Voino-Yasenetsky, Krasnoyarsk, Russia; 2 Department of Pediatrics, Krasnoyarsk State Medical University named after Prof. V.F. Voino-Yasenetsky, Krasnoyarsk, Russia; 3 Department of Children Infectious Diseases, Krasnoyarsk State Medical University named after Prof. V.F. Voino-Yasenetsky, Krasnoyarsk, Russia

**Keywords:** Lipopolysaccharide, Neuroinflammation, Blood-brain barrier, Lactate

## Abstract

**Background:**

Lipopolysaccharide (LPS) is one of the main constituents of the cell wall of gram-negative bacteria. As an endotoxin, LPS induces neuroinflammation, which is associated with the blood-brain barrier impairment. Lactate is a metabolite with some significant physiological functions within the neurovascular unit/blood-brain barrier (BBB). Accumulation of extracellular and cerebrospinal fluid lactate is a specific feature of bacterial meningitis. However, the role of lactate production, transport, and sensing by lactate receptors GPR81 in the pathogenesis of bacterial neuroinflammation is still unknown.

**Methods:**

In this study, we analyzed effects of LPS on the expression of GPR81 and MCT-1 and proliferation of cerebral endothelial cells in the BBB model in vitro. We used molecular profiling methods to measure the expression of GPR81, MCT-1, IL-1β, and Ki67 in the cerebral endothelium after treatment with different concentrations of LPS followed by measuring the level of extracellular lactate, transendothelial electric resistance, and permeability of the endothelial cell layer.

**Results:**

Our findings showed that exposure to LPS results in neuroinflammatory changes associated with decreased expression of GPR81 and MCT-1 in endothelial cells, as well as overproduction of IL-1β and elevation of lactate concentrations in the extracellular space in a dose-dependent manner. LPS treatment reduced JAM tight junction protein expression in cerebral endothelial cells and altered BBB structural integrity in vitro.

**Conclusion:**

The impairment of lactate reception and transport might contribute to the alterations of BBB structural and functional integrity caused by LPS-mediated neuroinflammation.

**Electronic supplementary material:**

The online version of this article (10.1186/s12974-018-1233-2) contains supplementary material, which is available to authorized users.

## Introduction

Bacterial meningitis is a life-threatening disease of the central nervous system (CNS), particularly, in children. Despite the progress in development of novel antibiotic-based treatment methods in early diagnosis of bacterial meningitis, the mortality and morbidity rates of this disease are still very high. Bacterial meningitis is recognized as the main cause of the top ten infection-associated death worldwide [1, 2]. The disease is an inflammation of meninges (arachnoid and pia mater) caused by the invasion of bacteria (*Haemophilus influenza*, *Neisseria meningitides*, streptococci, pneumococci) into the subarachnoid space [3]. Among these pathogens, *Neisseria meningitides* is the leading cause of the infection in children and young adults [4]. Lipopolysaccharide (LPS) is a major endotoxin found in Gram-negative bacteria with strong pro-inflammatory effects [5]. LPS is a major component of the *Neisseria meningitides* outer membrane, responsible for inflammatory responses in meningitis and sepsis [6, 7]. LPS is usually used for bacterial meningitis modeling in vivo and in vitro due to its well-known effects on immune cells and cerebral microvessel endothelial cells [8, 9].

Several pathogenic mechanisms lead to the development of brain injury in bacterial meningitis, including leukocyte transmigration and microglia activation. Such events cause cytotoxicity, neuronal cell death, and local production of pro-inflammatory cytokines and chemokines. Overall, these effects lead to acute neuroinflammation, remodeling of extracellular matrix, BBB breakdown, and progression of neurological deficits [10, 11].

BBB structural and functional integrity is controlled by various mechanisms, such as coordinated expression of tight junction and adherence junction proteins in brain microvessel endothelial cells (BMECs), functional activity of perivascular cells (pericytes, astrocytes), and complex intercellular interactions within the neurovascular unit (NVU) [12]. Loss of BBB integrity in bacterial meningitis can be primary (due to the direct effect of pathogens on NVU cells) or secondary (due to the overwhelming production of pro-inflammatory cytokines by microglial and astroglial cells, diminished metabolism of BMECs, and impaired reparative angiogenesis in cerebral microvessels) [13–16]. Interaction of bacteria with the brain endothelium alters the junctional machinery in BMECs, thereby resulting in neuroinflammation associated with pathological overproduction of cytokines with pro-inflammatory action (i.e., IL-1β, IL-6, TNFα, chemokines) and elevated permeability of the barrier [17]. Particularly, LPS induces a significant increase of BBB permeability by changing the RhoA signaling and cytoskeletal rearrangements in BMECs [18], stimulating the cyclooxygenase activity [19] and activating matrix metalloproteinase [20, 21]. Previous in vitro studies showed that LPS mediates the BBB breakdown at 24 h. This phenomenon is characterized by tight junction deregulation, reactive oxygen species production in BMECs, and cytokine production by activated microglia [22, 23]. However, some other reports suggested that BBB breakdown is not necessarily correlated with the pathogenesis of bacterial meningitis [2].

Inflammation in bacterial meningitis is accompanied by a prominent increase of lactate concentrations in cerebrospinal fluid (CSF) and can be used as a reliable criterion for differential diagnosis of bacterial and aseptic meningitis [24], and/or viral meningitis [25]. Local production and transport of lactate within the NVU supports integration and normal functions of BBB [12] whereas elevated concentrations of lactate might be associated with neuroinflammation and BBB impairment [26]. Within the neurovascular unit, lactate is produced by neurons, perivascular astroglial cells, or BMECs. Monocarboxylate transporters (MCTs) carry on the influx and efflux of lactate and contribute to effective metabolic coupling of NVU cells [27]. However, in some other cell types, inhibition of MCT-1 activity results in the suppression of angiogenesis [28]. GPR81 receptors transport lactate into BMECs. These receptors are expressed at luminal and abluminal membranes that act as metabolic sensors. As we demonstrated before, elevation of extracellular lactate or activation of GPR81 stimulate mitochondrial biogenesis and support angiogenic properties of BMECs in vitro [12, 29]. However, there are no data on the role of GPR81 or MCT-1 in the pathogenesis of BBB impairment in bacterial meningitis. Therefore, in this study, we analyzed the in vitro effects of LPS on the expression of GPR81 and MCT-1 in BMECs associated with neuroinflammation and BBB breakdown.

## Materials and methods

### Isolation and culture of BMECs within the BBB model in vitro

This study was performed on Wistar rats cells (*n* = 5 for isolation of astroglial and neuronal cells; *n* = 5 for isolation of cerebral endothelial cells). All experiments and animal use were conducted in compliance with the European Community Directive (2010/63/EC) and were approved by the Local Ethic Committee of the Krasnoyarsk State Medical University named after Prof. V.F. Voino-Yasenetsky. We isolated rat BMECs from P10 rats using the protocol of Liu et al. with minimal modifications [30]. Astroglial and neuronal cells were isolated from P4 neonatal rats according to a standard protocol [31]. The cells were cultured at 37 °C, 5% CO_2_ in Dulbecco’s modified Eagle’s medium (DMEM/F12 + 20% FBS, 3 mg/ml glucose, 0.58 mg/ml glutamine, 100 U/ml penicillin, 100 mg/ml streptomycin) in gelatin-coated vials (seeding density 1 × 10^5^ cell/ml). Culture medium was changed every 2–3 days. When the cells reached 90% confluence, they were separated by trypsinization and seeded in culture on gelatin-coated cups. Phase-contrast microscopy was continuously used to evaluate cell morphology (Additional file 1).

### Assessment of BBB integrity and marker expression

BBB model in vitro was reconstructed according to an original protocol described in details elsewhere [32]. Briefly, cells (endothelial, neuronal, and astroglial) were co-cultured in plates where neurons and astrocytes placed on wells bottom and endothelial cells placed on inserts while covered with semi-permeable membrane (incubated at 37 °С, 5% СО_2_).

Monitoring of BBB permeability was carried out using TEER measurement system known as an Epithelial Voltohmmeter (EVOM2) with chopstick electrode STX2 (World Precision Instrument, USA). Achieving a TEER of ≥ 120 Ω was used as a criterion to obtain BBB model with the permeability characteristics suitable for further studies.

Immunohistochemistry was used to assess the expression of GPR81 receptors, lactate transporter MCT-1, pro-inflammatory cytokine IL-1β, and cell proliferation marker Ki67 with appropriate primary and secondary antibodies. We used primary antibodies at 1:400 and secondary at 1:600 (Abcam, USA) followed by visualization with confocal laser microscopy (Olympus FV10i-W, Japan) and Olympus Fluoview software. The relative numbers of antigen-positive cells were counted in at least 10 fields.

Lactate extracellular concentrations were evaluated spectrophotometrically with the Lactate assay kit (Sigma-Aldrich, USA). Cell viability was tested with Trypan blue.

### Analysis of BMEC monolayer permeability for Lucifer yellow

Lucifer yellow (LY) assay allows measuring the endothelial layer permeability in vitro [22]. For transport experiments, all media were removed from the upper chamber of the insert and replaced with pre-warmed media with solutions of LY (50-μM working concentration). Samples were collected at 30, 60, and 90 min. At the end of each experiment (5 replicates), the concentration of the fluorescent compounds accumulated in the chamber was measured using a spectrofluorimeter (CM2203, “SOLAR”, Belarus) with excitation at 430/485 nm and emission at 535 nm. We used the following equation to measure the permeability coefficients:

$$ \mathrm{Permeability}\ \mathrm{coefficients}\ \left(\mathrm{Pe}\right)=\mathrm{dC}/\mathrm{dT}\times \mathrm{V}/\left(\mathrm{A}\times \mathrm{C}\right) $$

[V = sample volume, dC = concentration variations, dT = time variations, C = the initial concentration in the donor compartment, A = exposed surface (cellular monolayer in cm^2^).]

### LPS-induced inflammation in BBB model in vitro

We tested the effects of LPS on BMECs and BBB in vitro at 24 h of LPS exposure (*Escherichia coli* strain 0111:В4 LPS lot no L4391, Sigma, USA), concentrations 50 and 100 ng/ml (indicated as LPS_50_ and LPS_100_, respectively).

Statistical analysis was performed using the nonparametric data *χ*^2^ tests, Kruskal-Wallis test result with pairwise comparisons for the groups. *P* values below 0.05 were considered significant.

## Results and discussion

**Fig. 1:**
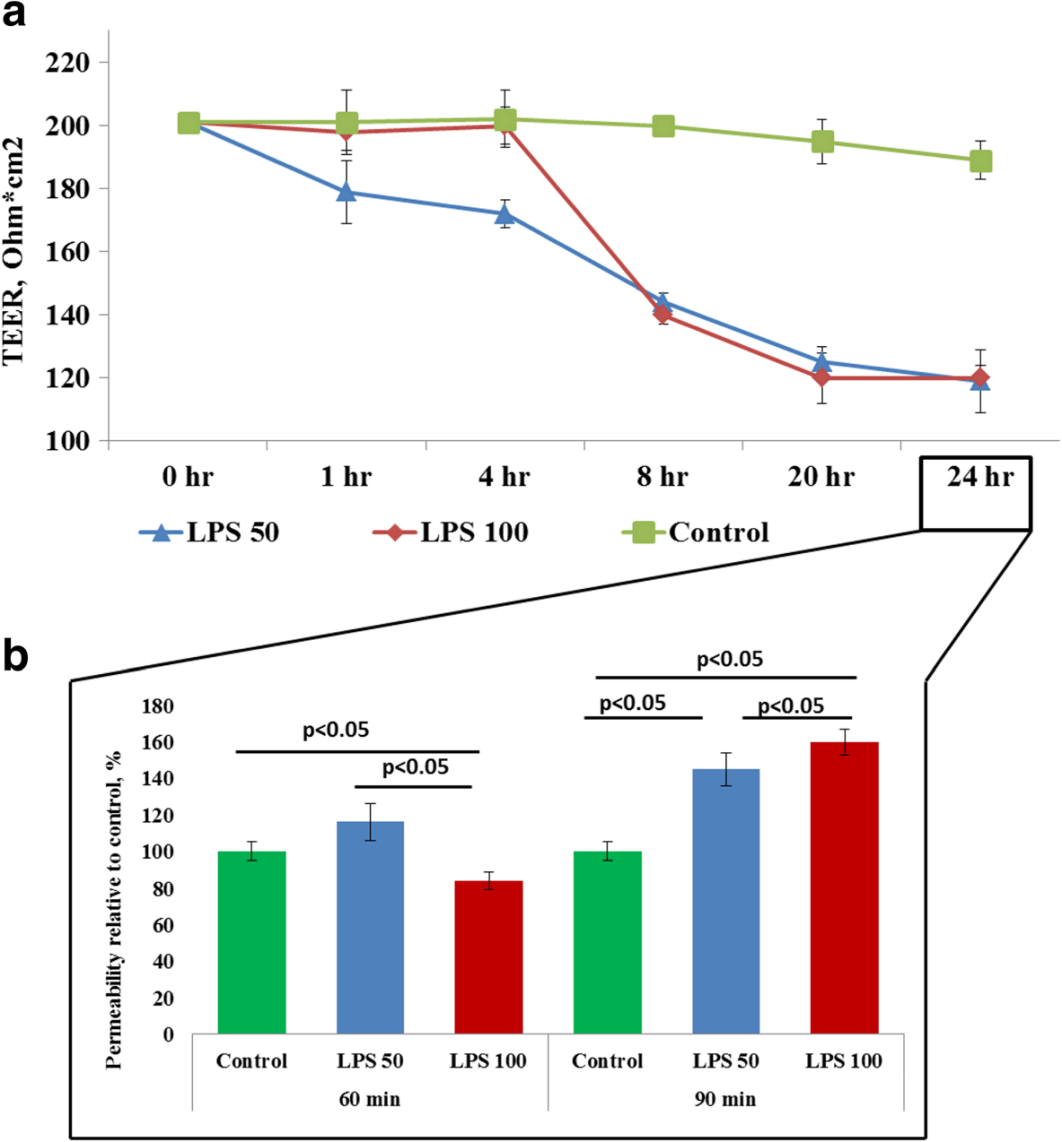
The effects LPS on TEER (**a**) and on the permeability (**b**) of blood-brain barrier model to Lucifer yellow (LY) in vitro. The cells of BBB were cultured with two concentrations of LPS, 50 ng/ml (blue lines; *n* = 14) and 100 ng/ml (red lines; *n* = 14) during 24 h. Control group (*n* = 14) is indicated in green color. The data represent the mean ± SD. *p* < 0.01

**Fig. 2:**
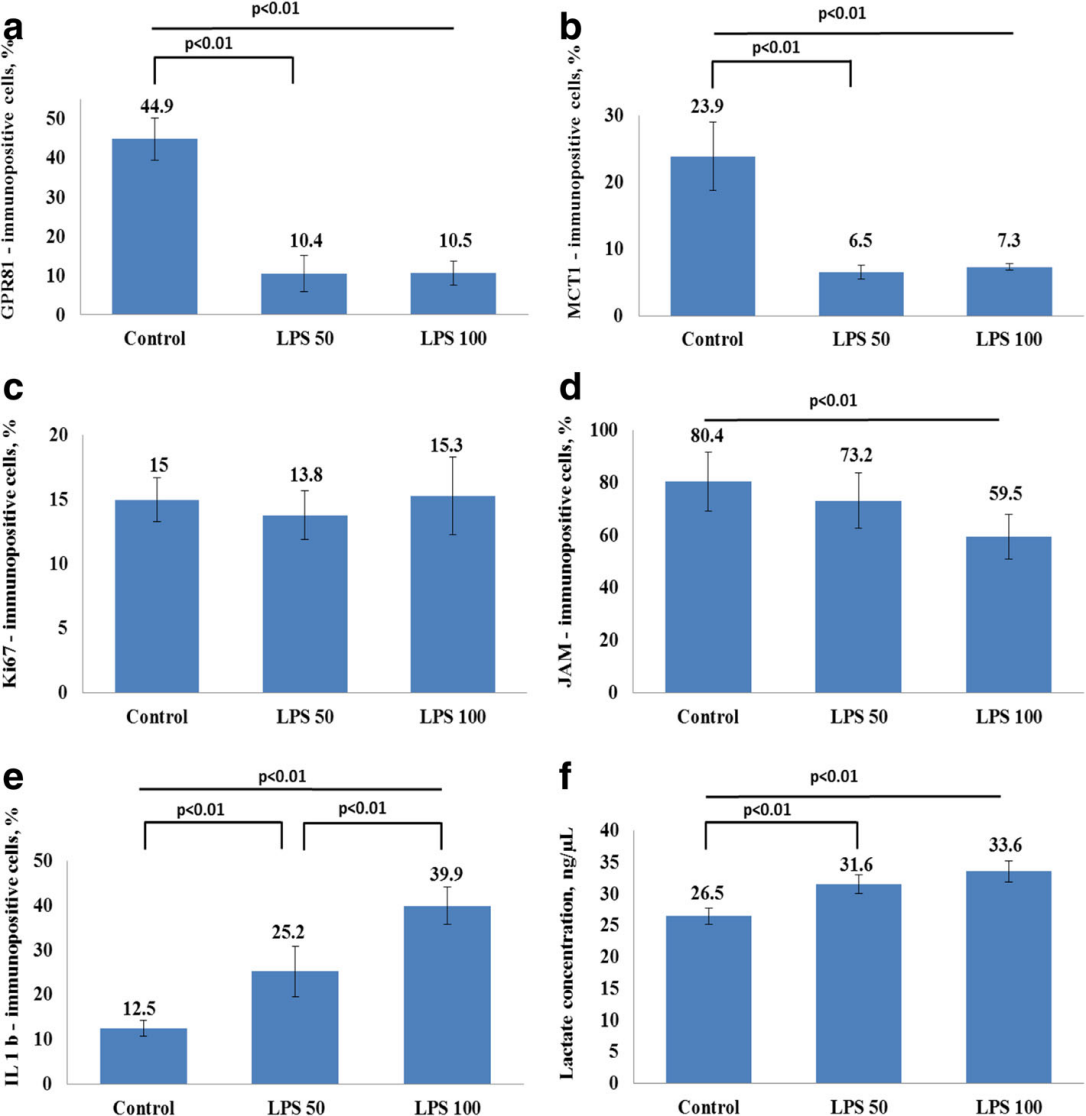
The effect of LPS on the expression of **a** GPR81, **b** MCT-1, **c** Ki67, **d** JAM, **e** IL-1β, and **f** lactate concentrations in the BBB in vitro model. The cells of BBB were cultured with LPS concentration 50 and 100 ng/ml during 24 h. The control group, *n* = 8; LPS_50_, *n* = 8; LPS_100_, *n* = 8. The data represent the mean ± SD. *p* < 0.01

In vitro exposure of rat BBB model to LPS in two tested concentrations during 24 h resulted in a progressive decrease of TEER and elevated permeability of the barrier in LY-test (Fig. 1a, b). Thus, LPS-induced breakdown was evident in BBB model. Incubation of BMECs (within the BBB model) with LPS exhibited a reduction in GPR81 expression after 24 h of incubation with no clear difference between two tested LPS concentrations (Fig. 2a). Similarly, expression of lactate transporter MCT-1 in brain microvessel endothelial cells was dramatically suppressed after incubation with LPS for 24 h (Fig. 2b). The observed changes in the expression of lactate transporters were associated with elevated concentrations of lactate in the culture medium at 24 h of BBB model exposure to LPS (Fig. 2f). However, these changes in extracellular lactate concentrations were not caused by cytolysis. We evaluated the viability of the cells in each group before measuring the expression of the target molecules and lactate levels in the medium. The average number of viable cells was comparable in all tested groups (95% in control versus 93% in LPS_50_-treated group and 94% in LPS_100_-treated group). Thus, elevated extracellular lactate is actively produced rather than passively released from damaged cells. The origin of lactate produced in the LPS-treated BBB model remains to be evaluated, i.e., astroglial production is rather possible.

**Fig. 3:**
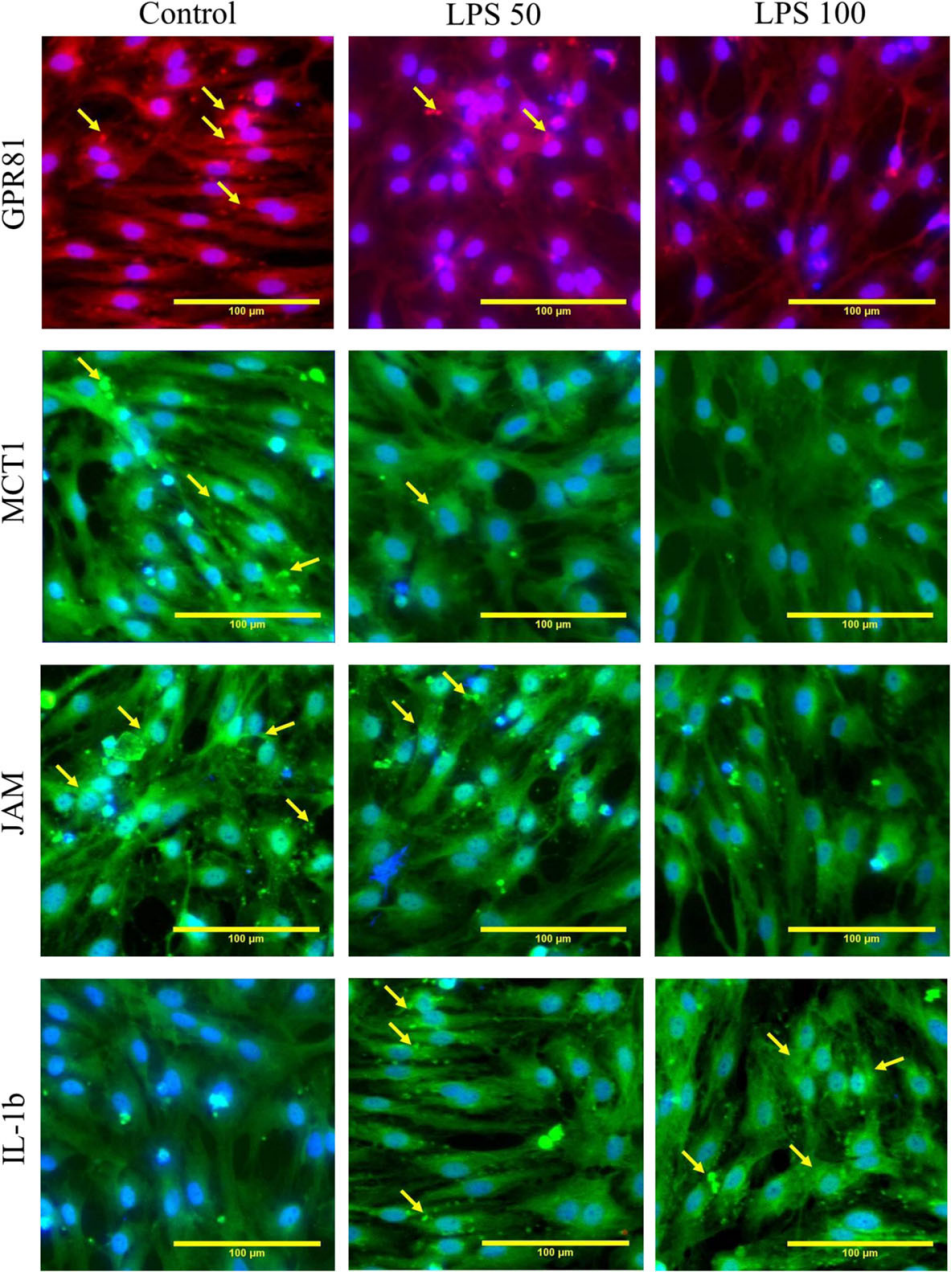
Microphotographs of endothelial monolayer in the model of BBB in vitro. The photos show GPR81, MCT-1JAM, and IL-1β expression after incubations of BMECs with LPS_50_ (*n* = 8), LPS_100_ (*n* = 8), or in the control group (*n* = 8). Scale bars 100 μm

We found a reduced expression of junctional adhesion molecule (JAM) proteins in BMECs (Fig. 2d) and elevated expression of pro-inflammatory cytokine IL-1β in BBB model at 24 h of LPS exposure (Fig. 2e). Thus, LPS-induced changes suggest establishment of a pro-inflammatory microenvironment, altered transport and reception of extracellular lactate, and deregulation of tight junction protein expression in BMECs (Fig. 3). Neuroinflammation and loss of BBB integrity are well-known triggers of BMEC proliferation [33]. Moreover, after being cultured within the BBB model, BMECs demonstrated visible angiogenic potential. Taking into the consideration that activation of glycolysis and lactate production is a prerequisite for BMEC activation and proliferation [34], we further tested the proliferative activity of BMECs which were exposed to LPS for 24 h. We assessed the expression of Ki67 protein in BMECs as a marker of cell proliferation. Moreover, altered expression of Ki67 was evident in human BMECs affected by *N*. *meningitis* [35]. We found that Ki67 expression in rat BMECs exposed to LPS was not significantly changed at 24 h (Fig. 2c). However, Ki67 expression in BMECs was significantly reduced at 72 h (16.5% Ki67-immunopositive cells in the control versus 10.6% in LPS_50_-treated group and 7.8% in LPS_100_-treated group (*p* < 0.01)). Thus, BBB breakdown and impaired transport and reception of lactate in BMECs were not associated with the stimulation of their proliferation in LPS-induced neuroinflammatuion, but rather resulted in long-lasting suppression of BMEC angiogenic potential.

Expression of GPR81 receptors have been previously reported in BMECs [36], but their role in the regulation of BBB integrity or neurovascular coupling is mainly unknown. Proposed involvement of GPR81 and MCT-1 in the pathogenesis of BBB alterations in cerebral malaria [26] suggests that similar mechanisms might be active in a case of acute meningitis. Excessive glycolysis in brain cells is a characteristic of bacterial meningitis [37]; thus, BMECs should be equipped with effective machinery for transporting and sensing elevated brain tissue lactate concentrations. However, observed changes in GPR81 and MCT-1 expression in LPS-treated cells of BBB in vitro could reflect impaired lactate clearance through the BBB in bacterial meningitis. Recently, we found that stimulation of GPR81 receptors in brain microvessel endothelial cells resulted in the stimulation of mitochondrial biogenesis [29] which is a prerequisite for cell proliferation and highly controlled permeability of endothelial cell layer [38, 39]. Therefore, reduced expression of lactate transporters and GPR81 in BMECs exposed to LPS could prevent lactate-mediated effects on mitochondrial dynamics, thus resulting in the suppression of endothelial metabolism. Given into the consideration that endothelial cells could use extracellular MCT-1-transported lactate as a “fuel” for mitochondrial respiration, we may propose that reduced expression of MCT-1 in LPS-treated BMECs within the BBB model would negatively affect their mitochondrial energy production and metabolic status. The latter could lead to impairment of tight junctions and barrier function of BMECs as well as suppression of angiogenesis. Thus, elevated production of lactate due to the presence of pro-inflammatory concentrations of LPS is associated with reduced transport and reception of lactic acid by endothelial cells, impaired lactate clearance mechanism, and loss of BBB structural integrity.

## Conclusions

Action of LPS in the BBB model in vitro results in endothelial layer impairment which is coupled to the accumulation of extracellular lactate and reduced ability of brain microvessel endothelial cells to sense or transport lactic acid. Reduced expression of GPR81 lactate receptors and MCT-1 lactate transporters in brain microvessel endothelial cells corresponds to the loss of BBB integrity and elevated permeability of the barrier in vitro.

## Additional file

Additional file 1: **Figure S4.** Phase contrast microphotograph (ZOE Fluorescent Cell Imager, Bio-rad, USA) of endothelial cells in the in vitro model of BBB, showing the monolayer status of cultured cells. Figure magnification × 20. Scale bar represents 100 μm. (DOCX 333 kb)

## Abbreviations

BBB: Blood-brain barrier
BMECs: Brain microvessel endothelial cells
CSF: Cerebrospinal fluid
GPR81: G protein-coupled receptor 81
IL-1β: Interleukin 1-beta
JAM: Junctional adhesion molecule
LPS: Lipopolysaccharide
MCT-1: Monocarboxylate transporter 1
NVU: Neurovascular unit
TEER: Transendothelial electric resistance

## References

[1] Yang R, Liu W, Miao L, Yang X, Fu J, Dou B, Cai A, Zong X, Tan C, Chen H, Wang X (2016). Induction of VEGFA and Snail-1 by meningitic Escherichia coli mediates disruption of the blood-brain barrier. Oncotarget.

[2] Kim KS (2008). Mechanisms of microbial traversal of the blood-brain barrier. Nat Rev Microbiol.

[3] Hoffman O, Weber RJ (2009). Pathophysiology and treatment of bacterial meningitis. Ther Adv Neurol Disord.

[4] Rouphael NG, Stephens DS (2012). *Neisseria meningitidis*: biology, microbiology, and epidemiology. Methods Mol Biol.

[5] West MA, Heagy W (2002). Endotoxin tolerance: a review. Crit Care Med.

[6] Piet JR, Zariri A, Fransen F, Schipper K, van der Ley P, van de Beek D, van der Ende A (2014). Meningitis caused by a lipopolysaccharide deficient Neisseria meningitidis. J Inf Secur.

[7] Ovstebo R, Aass HC, Haug KB, Troseid AM, Gopinathan U, Kierulf P, Berg JP, Brandtzaeg P, Henriksson CE (2012). LPS from Neisseria meningitidis is crucial for inducing monocyte- and microparticle-associated tissue factor activity but not for tissue factor expression. Innate Immun.

[8] O'Reilly T, Ostergaard C, Vaxelaire J, Zak O (2007). Systemic inflammation alters the inflammatory response in experimental lipopolysaccharide-induced meningitis. Clin Exp Immunol.

[9] Wispelwey B, Lesse AJ, Hansen EJ, Scheld WM (1988). Haemophilus influenzae lipopolysaccharide-induced blood brain barrier permeability during experimental meningitis in the rat. J Clin Invest.

[10] Scheld WM, Koedel U, Nathan B, Pfister HW (2002). Pathophysiology of bacterial meningitis: mechanism(s) of neuronal injury. J Infect Dis.

[11] Maisey HC, Hensler M, Nizet V, Doran KS (2007). Group B streptococcal pilus proteins contribute to adherence to and invasion of brain microvascular endothelial cells. J Bacteriol.

[12] Salmina AB, Kuvacheva NV, Morgun AV, Komleva YK, Pozhilenkova EA, Lopatina OL, Gorina YV, Taranushenko TE, Petrova LL (2015). Glycolysis-mediated control of blood-brain barrier development and function. Int J Biochem Cell Biol.

[13] Quagliarello VJ, Long WJ, Scheld WM (1986). Morphologic alterations of the blood-brain barrier with experimental meningitis in the rat. Temporal sequence and role of encapsulation. J Clin Invest.

[14] Barichello T, Lemos JC, Generoso JS, Cipriano AL, Milioli GL, Marcelino DM, Vuolo F, Petronilho F, Dal-Pizzol F, Vilela MC, Teixeira AL (2011). Oxidative stress, cytokine/chemokine and disruption of blood-brain barrier in neonate rats after meningitis by Streptococcus agalactiae. Neurochem Res.

[15] Norden DM, Trojanowski PJ, Villanueva E, Navarro E, Godbout JP (2016). Sequential activation of microglia and astrocyte cytokine expression precedes increased Iba-1 or GFAP immunoreactivity following systemic immune challenge. Glia.

[16] Skelly DT, Hennessy E, Dansereau MA, Cunningham C (2013). A systematic analysis of the peripheral and CNS effects of systemic LPS, IL-1beta, [corrected] TNF-alpha and IL-6 challenges in C57BL/6 mice. PLoS One.

[17] Lee CAA, Seo HS, Armien AG, Bates FS, Tolar J, Azarin SM (2018). Modeling and rescue of defective blood-brain barrier function of induced brain microvascular endothelial cells from childhood cerebral adrenoleukodystrophy patients. Fluids Barriers CNS.

[18] Dando SJ, Mackay-Sim A, Norton R, Currie BJ, St John JA, Ekberg JA, Batzloff M, Ulett GC, Beacham IR (2014). Pathogens penetrating the central nervous system: infection pathways and the cellular and molecular mechanisms of invasion. Clin Microbiol Rev.

[19] Banks WA, Gray AM, Erickson MA, Salameh TS, Damodarasamy M, Sheibani N, Meabon JS, Wing EE, Morofuji Y, Cook DG, Reed MJ (2015). Lipopolysaccharide-induced blood-brain barrier disruption: roles of cyclooxygenase, oxidative stress, neuroinflammation, and elements of the neurovascular unit. J Neuroinflammation.

[20] Rosenberg GA, Estrada EY, Mobashery S (2007). Effect of synthetic matrix metalloproteinase inhibitors on lipopolysaccharide-induced blood-brain barrier opening in rodents: differences in response based on strains and solvents. Brain Res.

[21] Ricci S, Grandgirard D, Wenzel M, Braccini T, Salvatore P, Oggioni MR, Leib SL, Koedel U (2014). Inhibition of matrix metalloproteinases attenuates brain damage in experimental meningococcal meningitis. BMC Infect Dis.

[22] Zhao Z, Hu J, Gao X, Liang H, Liu Z (2014). Activation of AMPK attenuates lipopolysaccharide-impaired integrity and function of blood-brain barrier in human brain microvascular endothelial cells. Exp Mol Pathol.

[23] Gresa-Arribas N, Vieitez C, Dentesano G, Serratosa J, Saura J, Sola C (2012). Modelling neuroinflammation in vitro: a tool to test the potential neuroprotective effect of anti-inflammatory agents. PLoS One.

[24] Li Y, Zhang G, Ma R, Du Y, Zhang L, Li F, Fang F, Lv H, Wang Q, Zhang Y, Kang X (2015). The diagnostic value of cerebrospinal fluids procalcitonin and lactate for the differential diagnosis of post-neurosurgical bacterial meningitis and aseptic meningitis. Clin Biochem.

[25] Giulieri S, Chapuis-Taillard C, Jaton K, Cometta A, Chuard C, Hugli O, Du Pasquier R, Bille J, Meylan P, Manuel O, Marchetti O (2015). CSF lactate for accurate diagnosis of community-acquired bacterial meningitis. Eur J Clin Microbiol Infect Dis.

[26] Mariga ST, Kolko M, Gjedde A, Bergersen LH (2014). Lactate transport and receptor actions in cerebral malaria. Front Neurosci.

[27] Lottes RG, Newton DA, Spyropoulos DD, Baatz JE (2015). Lactate as substrate for mitochondrial respiration in alveolar epithelial type II cells. Am J Physiol Lung Cell Mol Physiol.

[28] Vegran F, Boidot R, Michiels C, Sonveaux P, Feron O (2011). Lactate influx through the endothelial cell monocarboxylate transporter MCT1 supports an NF-kappaB/IL-8 pathway that drives tumor angiogenesis. Cancer Res.

[29] Khilazheva ED, Pisareva NV, Morgun AV, Boitsova EB, Taranushenko TE, Frolova OV, Salmina AB (2017). Activation of GPR81 lactate receptors stimulates mitochondrial biogenesis in cerebral microvessel endothelial cells. Ann Clin Exp Neurol.

[30] Liu Y, Xue Q, Tang Q, Hou M, Qi H, Chen G, Chen W, Zhang J, Chen Y, Xu X (2013). A simple method for isolating and culturing the rat brain microvascular endothelial cells. Microvasc Res.

[31] Schildge S, Bohrer C, Beck K, Schachtrup C. Isolation and culture of mouse cortical astrocytes. J Vis Exp. 2013;(71) 10.3791/50079. 10.3791/50079 PMC3582677 23380713

[32] Khilazheva ED, Boytsova EB, Pozhilenkova EA, Solonchuk YR, Salmina AB (2015). Obtaining a three-cell model of a neurovascular unit in vitro. Cell and Tissue Biology.

[33] Welser JV, Li L, Milner R (2010). Microglial activation state exerts a biphasic influence on brain endothelial cell proliferation by regulating the balance of TNF and TGF-beta1. J Neuroinflammation.

[34] Stapor P, Wang X, Goveia J, Moens S, Carmeliet P (2014). Angiogenesis revisited—role and therapeutic potential of targeting endothelial metabolism. J Cell Sci.

[35] Oosthuysen WF, Mueller T, Dittrich MT, Schubert-Unkmeir A (2016). Neisseria meningitidis causes cell cycle arrest of human brain microvascular endothelial cells at S phase via p21 and cyclin G2. Cell Microbiol.

[36] Lauritzen KH, Morland C, Puchades M, Holm-Hansen S, Hagelin EM, Lauritzen F, Attramadal H, Storm-Mathisen J, Gjedde A, Bergersen LH (2014). Lactate receptor sites link neurotransmission, neurovascular coupling, and brain energy metabolism. Cereb Cortex.

[37] Guerra-Romero L, Tauber MG, Fournier MA, Tureen JH (1992). Lactate and glucose concentrations in brain interstitial fluid, cerebrospinal fluid, and serum during experimental pneumococcal meningitis. J Infect Dis.

[38] Doll DN, Hu H, Sun J, Lewis SE, Simpkins JW, Ren X (2015). Mitochondrial crisis in cerebrovascular endothelial cells opens the blood-brain barrier. Stroke.

[39] Tang X, Luo YX, Chen HZ, Liu DP (2014). Mitochondria, endothelial cell function, and vascular diseases. Front Physiol.

