# Supplementary material for: The inhibitory effect of LPS on the expression of GPR81 lactate receptor in blood-brain barrier model in vitro
Source: J Neuroinflammation. 2018 Jul 4;15:196. doi: 10.1186/s12974-018-1233-2 (PMC6030740; doi:10.1186/s12974-018-1233-2)
Supplement: Supplementary file 1 — Figure S4. Phase contrast microphotograph (ZOE Fluorescent Cell Imager, Bio-rad, USA) of endothelial cells in the in vitro model of BBB, showing the monolayer status of cultured cells. Figure magnification × 20. Scale bar represents 100 μm. (DOCX 333 kb) [file 12974_2018_1233_MOESM1_ESM.docx]

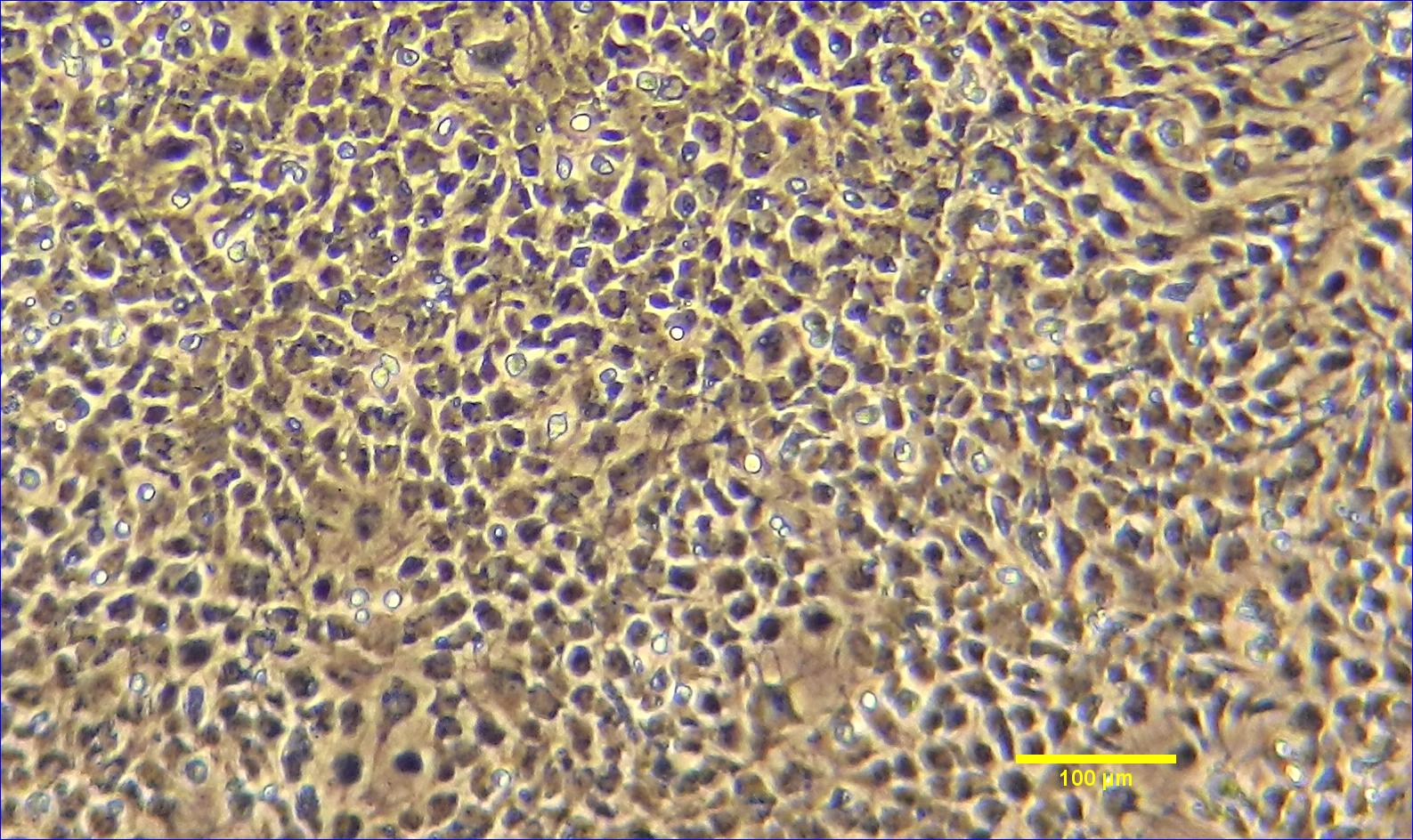


**Figure S4.** Phase contrast microphotograph (ZOE Fluorescent Cell Imager, Bio-rad, USA) of endothelial cells in the *in vitro* model of BBB, showing the monolayer status of cultured cells. Figure magnification: 20 X. Scale bar represents 100 µm.
